# Real-Time Quaking- Induced Conversion Assays for Prion Diseases, Synucleinopathies, and Tauopathies

**DOI:** 10.3389/fnagi.2022.853050

**Published:** 2022-03-10

**Authors:** Sarah Vascellari, Christina D. Orrù, Byron Caughey

**Affiliations:** ^1^Department of Biomedical Sciences, University of Cagliari, Cagliari, Italy; ^2^Laboratory of Persistent Viral Diseases (LPVD), Rocky Mountain Laboratories, National Institute of Allergy and Infectious Diseases (NIAID), National Institute of Health (NIH), Hamilton, MT, United States

**Keywords:** RT-QuIC assay, diagnosis, seeding activity, prion disorders, synucleinopathies, tauopathies

## Abstract

Prion diseases, synucleinopathies and tauopathies are neurodegenerative disorders characterized by deposition of abnormal protein aggregates in brain and other tissues. These aggregates consist of *misfolded* forms of prion, α-synuclein (αSyn), or tau proteins that cause neurodegeneration and represent hallmarks of these disorders. A main challenge in the management of these diseases is the accurate detection and differentiation of these abnormal proteins during the early stages of disease before the onset of severe clinical symptoms. Unfortunately, many clinical manifestations may occur only after neuronal damage is already advanced and definite diagnoses typically require post-mortem neuropathological analysis. Over the last decade, several methods have been developed to increase the sensitivity of prion detection with the aim of finding reliable assays for the accurate diagnosis of prion disorders. Among these, the real-time quaking-induced conversion (RT–QuIC) assay now provides a validated diagnostic tool for human patients, with positive results being accepted as an official criterion for a diagnosis of probable prion disease in multiple countries. In recent years, applications of this approach to the diagnosis of other prion-like disorders, such as synucleinopathies and tauopathies, have been developed. In this review, we summarize the current knowledge on the use of the RT-QuIC assays for human proteopathies.

## Introduction

Several neurodegenerative proteinopathies are characterized by the conformational change of specific native proteins, which form intra- or extracellular pathological aggregates in particular brain regions, leading to neurodegeneration and progression of clinical symptoms. Prion protein (PrP), α-synuclein (αSyn) and tau aggregates represent hallmarks of prion disorders, synucleinopathies and tauopathies, respectively. Although the proteins involved and the resultant clinical symptoms vary, these proteopathies share a common prion-like seeded propagation mechanism for the causative aggregates.

These aggregates may be present in biological fluids and tissues prior to the onset of clinical signs, making them attractive biomarkers for the early diagnosis of the associated neurodegenerative disorders. However, their detection can be challenging due to low concentrations in accessible biospecimens. This, combined with heterogeneous and often overlapping clinical manifestations especially in early stages of disease, complicates accurate *intra vitam* diagnosis of many proteopathies. Accordingly, although brain electrophysiological and imaging findings in combination with non-specific fluid biomarkers can be useful in the diagnostic process, definitive diagnoses often depend on *post-mortem* pathological examination of brain tissues for specific protein deposits.

Over the last decade ultrasensitive real-time quaking- induced conversion (RT-QuIC) seed amplification assays (SAAs) have been developed for proteopathic aggregates based on their seeding activities (Wilham et al., 2010; Atarashi et al., 2011). RT-QuIC allows the specific detection of as little as femptogram or attogram amounts of proteopathic seeds in biospecimens, opening the way for improved *intra vitam* detection and diagnoses for various prion-like neurodegenerative disorders. Although initially developed and clinically implemented for the diagnosis of prion diseases, the RT-QuIC platform has been adapted more recently to the detection of seeds of αSyn and tau associated with synucleinopathies [e.g., Parkinson’s disease (PD), dementia with Lewy bodies (DLB), multiple system atrophy (MSA)] and tauopathies [e.g., Alzheimer’s disease (AD), chronic traumatic encephalopathy (CTE), Pick’s disease (PiD), Progressive supranuclear palsy (PSP), and corticobasal syndrome (CBS)]. In this review, we summarize recent advances in the application of RT-QuIC assays to the study and diagnosis of these human proteopathies.

## Prion Disorders

Prion diseases (or transmissible spongiform encephalopathies) involve the ordered assembly of PrP molecules into highly transmissible fibrils (Hoyt et al., 2021; Kraus et al., 2021a,b; Manka et al., 2021). These diseases affect humans [e.g., Creutzfeldt-Jakob disease (CJD), Gerstmann-Sträussler-Scheinker (GSS), Fatal familial insomnia (FFI)] and many other mammals (e.g., Scrapie, Bovine Spongiform Encephalopathy, Chronic wasting disease). The normal cellular PrP isoform (PrP^C^) has a predominantly α-helical C-terminal domain and a flexible N-terminal domain (Wuthrich and Riek, 2001). Structures of the infectious forms of PrP (generically called PrP^Sc^) solved to date are amyloid fibrils in which the PrP monomers are assembled with a parallel, in-register intermolecular β-sheet (PIRIBS) core, providing a precise strain-dependent template for seeded prion growth (Hoyt et al., 2021; Kraus et al., 2021a,b; Manka et al., 2021). The recruitment of PrP molecules into the PrP^Sc^ fibril requires a complete refolding of PrP^C^ (Kraus et al., 2021a). Fragmentation of the elongating fibril increases the number of prion particles, and their respective templating surfaces at the fibril ends (Kraus et al., 2021a; Meisl et al., 2021). Thereby, PrP^Sc^ accumulates and spreads in host tissues, most notably and consequentially in the brain.

### Prion Real-Time Quaking- Induced Conversion

**FIGURE 1:**
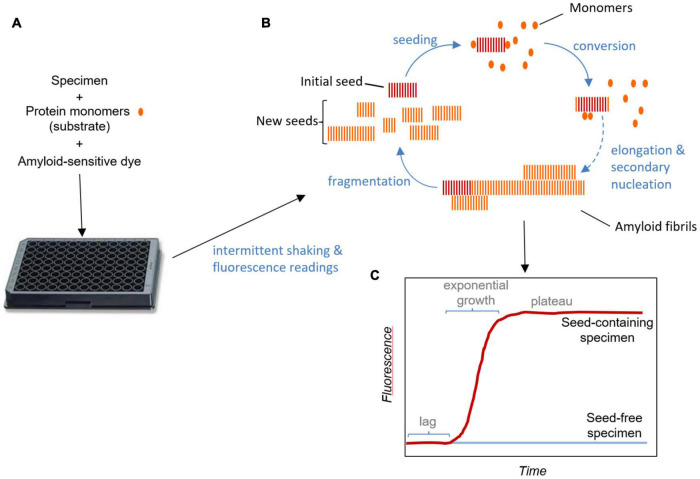
Real-time quaking- induced conversion assay schematic. **(A)** Test specimens are added to multi-well plates along with solutions of substrate protein monomers and an amyloid sensitive dye such as thioflavin T. The plates are subjected to intermittent shaking and fluorescence readings in a temperature-controlled plate-reader. **(B)** Amplification cycle: Seeds in the specimen bind monomers and induce their conformational conversion as they are recruited into the growing fibril. Secondary nucleation may occur due to assembly and conversion of monomers on the sides of fibrils, contributing, along with fragmentation, to the generation of new seeding surfaces. New seeding surfaces are critical in obtaining exponential growth kinetics. **(C)** Fluorescence readout: Binding of the dye to amyloid fibrils enhances its fluorescence. In the absence of pre-existing seeds in the specimen, amyloid fibril formation requires spontaneous nucleation, which, under conditions optimized for these assays at least, is much slower than the growth and fragmentation of pre-existing seeds. In reactions provided with pre-existing seeds, the lag corresponds to the phase in which seeds are growing at sub-detectable levels. The fluorescence plateaus once the available monomer is consumed. Sometimes fluorescence can even decrease after peaking, an effect that is likely due to consolidation or redistribution of fibrils in the well in a way that affects the fluorescence measurement (not illustrated).

A main goal in the management of prion disorders is to detect the prion seeds as early as possible in infected individuals. SAAs like RT-QuIC amplify otherwise undetectable levels of prion seeds in diagnostic specimens (Saborio et al., 2001; Atarashi et al., 2007, 2011; Colby et al., 2007; Wilham et al., 2010). Like the preceding amyloid seeding assay (Colby et al., 2007), the prototypic prion RT-QuIC assay developed by the Atarashi and Caughey labs (Wilham et al., 2010; Atarashi et al., 2011) amplifies the presence of prion seeds by up to a trillion-fold in shaken, multiwell plates with a fluorescence readout (Figure 1). In these reactions, prions in the sample seed conversion of a vast excess of recombinant prion protein (rPrP; the reaction substrate) into fibrils, which enhances the fluorescence of Thioflavin T (ThT). RT-QuIC reactions have been adapted to virtually all known prions of mammals [(Orru et al., 2015) and dozens of other publications]. The reaction plate (96–384-well) is subjected to cycles of shaking and rest at a controlled temperature in a fluorescence plate reader. As is typical of the seeded polymerization of amyloid fibrils generally, positive reactions can be separated into three phases: The *lag phase* represents the time until the fluorescence crosses a predetermined threshold for positivity. During this time sub-detectable growth of the prion seeds is occurring. The ensuing *exponential growth phase* is characterized not only by fibril elongation, but also the generation of new seeding surfaces by fibril fragmentation and/or secondary nucleation. The final *plateau phase* reflects exhaustion of the available rPrP substrate. In some reactions, the fluorescence can decline after reaching a maximum. This might be due either to bundling of fibril products in a way that displaces or quenches ThT fluorescence, or to repositioning of fibrils in the reaction well away from the optical beam paths. It does not seem to be due to degradation of ThT (Orru et al., 2016).

The main advantages of RT-QuIC relative to predecessors are high sensitivity and specificity; use of bacterially expressed PrP^C^ as a substrate; easily replicable agitation; high through-put reaction format; fluorescence readout; and the ability to continuously monitor the progress of reactions. Methodological improvements to increase the reaction sensitivity for the detection of low levels of prion seeds in accessible specimens have included manipulating the rPrP substrate, salts, sodium dodecyl sulfate (SDS), shaking, and/or temperature. Through such manipulations, RT-QuIC assays have been adapted by many laboratories to a wide range of biospecimens [e.g., brain homogenates (BH), cerebrospinal fluid (CSF), plasma, olfactory mucosa (OM), skin] and prions, from the most common sCJD to rare genetic forms in humans. These advances have enabled continuous progress toward RT-QuIC applicability in clinical practice.

The first human application of RT-QuIC used full length human rPrP (Hu 23–231, 0.06–0.1 g/L) as substrate, 500 mM NaCl, circular shaking conditions (30, 120 s rest), an incubation temperature of 37°C and a 90-h reaction time (Atarashi et al., 2011). This allowed detection of sCJD seeds in brain homogenate dilutions with as little as femtogram amounts of PrP^CJD^. Analysis of CSF samples gave a diagnostic sensitivity of 80% and a specificity of 100%. A second study showed an increase of sensitivity up to 91% in the detection of sCJD seeds from CSF samples using full length hamster rPrP (Ha 23–231, 0.1 g/L) substrate, 300 mM NaCl, double orbital shaking conditions (60 s quaking 600 rpm, 60 s rest) and an incubation temperature of 42°C (McGuire et al., 2012). Same reaction conditions were then reproduced in several laboratories from Europe, United Kingdom, United States, Australia, Canada to Japan, in tests of large panels of CSF samples from sCJD patients. The studies showed sensitivities between 73 and 100% and specificities of 98–100% (Orru et al., 2014; Bongianni et al., 2017; Lattanzio et al., 2017; Green, 2019; Candelise et al., 2020). The high reproducibility of this test was shown across multiple laboratories despite the use of different substrates, sample storage conditions, and instrumentation. Further studies showed similar results in tests of CSF samples from genetic forms of GSS, FFI and gCJD with diagnostic sensitivities of 90, 83.3, and 81.8%, using the full length human rPrP (Hu 23–231) (Sano et al., 2013), and chimeric hamster-sheep rPrP (Ha 14–128–Sheep 141–234) for gCJD (100% sensitivity, 99% specificity) (Cramm et al., 2015), respectively. These studies provided strong evidence that RT-QuIC analysis of CSF can provide high diagnostic accuracy for different prion diseases.

### Second Generation Prion Real-Time Quaking- Induced Conversion

A more rapid and sensitive “second generation” RT-QuIC assay for human diagnostics introduced the use of the truncated hamster rPrP (90–231) as substrate, 0.002% SDS, 700 rpm shaking and an incubation temperature of 55°C. This assay detected sCJD seeds in CSF samples with a diagnostic sensitivity of (95.8%) and specificity (100%) and a reaction time of 14–55 h. Further applications of second generation RT-QuIC to a large panel of CSFs from patients with different prion diseases confirmed high sensitivity (70–100%) and specificity (98–100%) and extended its use across several prion diseases and laboratories (Bongianni et al., 2017; Foutz et al., 2017; Franceschini et al., 2017; Groveman et al., 2017; Fiorini et al., 2020; Rhoads et al., 2020). In addition, several studies using first and second generation RT-QuIC assays reported variations in reaction sensitivity in distinct clusters of sCJD patients depending on the polymorphism at codon 129 of the PrP gene and the type of PrP^Sc^, with higher sensitivities in MM1/MV1 cases (89–96%) than in MV2 (81–89%) and VV2 (78–100%) cases. Lower sensitivities were obtained in the atypical subtypes (e.g., MM2 and VV1) (Green, 2019).

Comparative studies on CSF protein biomarkers of neurodegeneration showed that prion RT-QuIC had higher diagnostic accuracy than 14-3-3 and tau tests for prion diseases (McGuire et al., 2016; Foutz et al., 2017; Lattanzio et al., 2017; Hermann et al., 2018; Fiorini et al., 2020). Attempts have also been made to use blood as a diagnostic specimen, given that it is more accessible than CSF. To overcome the problem that RT-QuIC reactions can be inhibited by components found in plasma or whole blood (e.g., red and white cells), an immunoprecipitation step with the PrP^Sc^-reactive 15B3 antibody was combined with RT-QuIC (Orru et al., 2011). This approach, called “enhanced QuIC” (eQuIC), was able to detect very low levels of PrP^CJD^ (≥1 attogram) in human plasma spiked with variant CJD BH dilutions and endogenous PrP^Sc^ in plasma from wild-type rodents and anchorless PrP transgenic mice infected with different prion strains (Orru et al., 2011, 2012; Vascellari et al., 2012). Although these results were promising as a proof-of-principle, use of the antibody-15B3 has been hindered by high cost and inconsistent performance. A further milestone was achieved when Orru et al. (2014) demonstrated the applicability of RT-QuIC to OM samples obtained from nasal brushings of sCJD patients (Bongianni et al., 2017). This initial study showed that OM samples can provide improvements in diagnostic sensitivity (98%), without losing specificity (100%), using a sample that can be obtained by a brushing procedure that is less invasive than lumbar puncture. A recent prospective study showed that the combination of CSF- and OM-based RT-QuIC assays can provide an overall diagnostic accuracy of 100% compared to 14-3-3 and tau tests (85% of sensitivity; 45% and 70% of specificity, respectively). This improves prospects for early and potentially definite *intra vitam* diagnosis of sCJD (Fiorini et al., 2020). Recently, two independent studies have extended the use of RT-QuIC to skin biopsies from variant and sCJD patients (Orru et al., 2017; Mammana et al., 2020), showing a sensitivity of 89% and specificity of 100%. This work indicates potential applicability of skin-based RT-QuIC diagnosis in clinical practice.

Based on these and other successful applications this ultrasensitive assay in prion seed detection, the European Centre for Disease Prevention and Control (ECDC) and the US Centers for Disease Control and Prevention (CDC) have introduced RT-QuIC analysis of CSF or other tissues as a criterion for diagnosis of probable sCJD.

## Synucleinopathies

Like prion diseases, synucleinopathies are a heterogeneous group of neurodegenerative syndromes with different clinical phenotypes, including PD, DLB, and MSA. Less common syndromes such as pure autonomic failure (PAF) and idiopathic rapid eye movement (REM) sleep behavior disorder (iRBD) are considered prodromal syndromes of MSA and Lewy bodies disorders (Iranzo et al., 2009; Hogl et al., 2018). These diseases are characterized by neuronal (e.g., PD, DLB) or glial cytoplasmic (e.g., MSA) inclusions composed mainly of αSyn. Normal αSyn is often an intrinsically disordered monomeric protein characterized by low hydrophobicity and high net charge (Lee et al., 2007). The structure of αSyn can be divided into three overall domains (N-terminal; non-amyloid component; and C-terminal) (Jao et al., 2004). Similar to PrP, native αSyn exists in a non-pathological largely disordered state (Burre et al., 2018), that under pathological conditions can self-aggregate and spread in a prion-like manner to accumulate as specific strain-like conformers of PIRIBS-based amyloid fibrils in host tissues (Tuttle et al., 2016; Schweighauser et al., 2020). Thus, native αSyn is required as substrate for the self-aggregation and self-propagation process (Volpicelli-Daley et al., 2011; Dunning et al., 2013; Masuda-Suzukake et al., 2014).

### α Synuclein Real-Time Quaking- Induced Conversion

Successes with prion RT-QuIC assays encouraged the development of analogous αSyn RT-QuIC (Fairfoul et al., 2016; Groveman et al., 2018), or similar αSyn PMCA (Shahnawaz et al., 2017), assays. As a clarification of terminology, we note that in contrast to RT-QuIC assays, the original prion PMCA (protein misfolding cyclic amplification) assay platform involved sonication of individual reaction tubes containing PrP^C^ in brain homogenates (Saborio et al., 2001) or recombinant PrP^C^ as the substrate (Atarashi et al., 2011) with Western blot-based detection of reaction products. However, the assay named “αSyn PMCA” is more akin RT-QuIC assays with reactions performed in multi-well plates with intermittent shaking and fluorescence readings. In any case, these assays have now been applied to the range of αSynucleinopathies mentioned above, and to a variety of biospecimens, including brain, CSF, OM, skin and submandibular gland (Fairfoul et al., 2016; Groveman et al., 2018; Bongianni et al., 2019; De Luca et al., 2019; van Rumund et al., 2019; Manne et al., 2020a,b).

The first-reported αSyn RT-QuIC assay was from Alison Green’s group (Fairfoul et al., 2016) and included human recombinant αSyn as a substrate and double orbital shaking in the presence of zirconia/silica beads (1 min quaking at 200 rpm/14 min rest) at 30°C. Analysis of an initial sample panel demonstrated to detection of αSyn seeds in brain and CSF samples from PD and DLB cases with an impressive sensitivity of 95 and 92%, respectively, and an overall specificity of 100%. Other studies reproducing similar conditions confirmed and extended evidence for high sensitivity and specificity in the detection of αSyn seeds in CSF samples from patients with distinct synucleinopathies, such as PD (84–96% sensitivity, 82–98% specificity) (Kang et al., 2019; van Rumund et al., 2019), DLB (92.6 sensitivity, 95.9% specificity) (Bongianni et al., 2019), MSA and other synucleinopathies (35–86% sensitivity, 98% specificity) (van Rumund et al., 2019). The same approach was applied to the detection of α-synuclein seeds in CSF from genetic cases of PD linked to the mutation in LRRK2 (Leucine-rich repeat kinase) gene with a sensitivity of 40% and specificity of 80% (Garrido et al., 2019).

Another important study showed that this protocol was able to detect α- synuclein seeds in iRBD, with a sensitivity of 90% and specificity of 78% (Iranzo et al., 2021). Given that iRBD is considered prodromal PD, this result has important implications for applicability of αSyn RT-QuIC assay in the early diagnosis of synucleinopathies.

A second assay αSyn PMCA was developed by Shahnawaz et al. (2017). Unlike the original PMCA prion amplification method that required sonication of reaction tubes, αSyn PMCA, like RT-QuIC, uses intermittent shaking of multiwell plates (1 min at 500 rpm/29 min rest) and ThT fluorescence readout. Other characteristics included the use of recombinant αSyn substrate (human full-length), in PIPES reaction buffer supplemented with 500 mM NaCl, and an incubation temperature at 37°C for ∼16 days. Two studies showed that αSyn PMCA assay detected αSyn seeding activity in CSF samples from patients with different synucleinopathies with sensitivities between 88.5 and 95% for PD patients, and 80 and 85% for MSA cases, and specificities between 94 and 100% (Shahnawaz et al., 2017, 2020). Furthermore, the same authors reported that distinct fluorescent profiles of αSyn PMCA reactions could be used to discriminate αSyn seeds in CSF samples from PD and MSA cases (Shahnawaz et al., 2020).

A third protocol was developed by Groveman et al. (2018). The main differences concerning the use of a mutant human recombinant αSyn (K23Q Hu) as substrate, 0.0015% SDS, double orbital shaking with silica beads (1 min quaking at 400 rpm/1 min rest) at 42°C. This protocol allowed much more rapid detection of seeds within 1–2 days (vs. 5–13 days for the prior αSyn RT-QuIC and PMCA assays), and initial testing of CSF samples from PD and DLB cases showed similarly high sensitivity (92 and 94%) and an overall specificity of 100%. Further testing of larger and broader CSF panels from PD (Manne et al., 2019; Rossi et al., 2020; Orru et al., 2021; Russo et al., 2021), DLB, iRBD, and PAF patients gave sensitivities between 93and 100% and specificities between 84 and 98% (Rossi et al., 2020). As with the Green assay, the Groveman assay failed to detect αSyn seeds in CSF from most MSA cases, consistent with MSA seeds being conformationally distinct from those Lewy body disorders.

Studies have also shown the applicability of αSyn RT-QuIC to OM samples with a sensitivity of 55.5% in PD cases, an increased sensitivity of 81.8% in MSA samples, and a specificity of 84.4% (De Luca et al., 2019). Two independent αSyn real-time quaking-induced conversion (RT–QuIC) studies detected seeding activity in the *post-mortem* skin samples from different synucleinopathies, such as PD, DLB, and MSA, with 93–94% sensitivity and a 93–98% specificity (Manne et al., 2020b; Wang et al., 2020).

Finally, a recent study has revealed seeding activity in *post-mortem* submandibular gland from PD cases by αSyn RT–QuIC with an overall sensitivity and specificity of 100% (Manne et al., 2020a). Such results suggest that αSyn seeds might also be detected in saliva samples from PD patients.

## Tauopathies

Tauopathies such as the previously mentioned AD, CTE, PiD, PSP, and CBS represent a wide range of clinical and neuropathological phenotypes (Ferrer et al., 2014). However, the common thread in these diseases is the accumulation and deposition of various types of tau aggregates in brain. Tau is a neuronal and glial protein that binds microtubules through microtubule-binding repeat domains near the C-terminus, and thereby regulates microtubule stability and axonal transport (Silva and Haggarty, 2020). Similar to αSyn, native tau, which is encoded by *MAPT* gene, is classified as intrinsically disordered due to the absence of a well-defined tertiary structure. This feature makes tau particularly prone to conformational changes including assembly into high β-sheet-rich structures (Avila et al., 2016). In pathological conditions tau undergoes post-translational modifications (e.g., phosphorylation, acetylation, ubiquitination), which promote and/or are potentiated by the formation of toxic oligomers, fibrils, and aggregates known as neurofibrillary tangles (NFTs; Alquezar et al., 2020). As a result of differential splicing of the mRNAs encoded by the *MAPT* gene, adult humans normally express six isoforms containing either three (3R) or four (4R) microtubule-binding repeats, and zero, one or two N domains (i.e., 0N3R, 1N3R, 2N3R, 0N4R, 1N4R, 2N4R tau isoforms). All of these tau isoforms can be incorporated into fibrillar inclusions, but isoform usage differs among tauopathies, consistent with the existence of distinct strains of tau fibrils [reviewed in Stahlberg and Riek (2021)]. This variability in tau fibril strains contributes to the phenotypic heterogeneity among tauopathies and the associated complexities of diagnosis. Adding to these complexities is the fact that tau deposits can be classified as either primary or secondary neuropathologies; in the latter case, tau deposits co-exist with deposits of other proteins. PiD, PSP, and CBS are considered as primary tauopathies characterized by the deposition of 3R (e.g., PiD) or 4R tau isoforms (e.g., PSP, CBS). AD represents a secondary tauopathy associated with the presence of β-amyloid plaques and the deposition of both 3R and 4R tau isoforms NFTs.

### Tau Real-Time Quaking- Induced Conversion

Consistent with previous RT-QuIC assays, Tau RT-QuIC assays exploit the ability of pathological tau aggregates to seeds the assembly of recombinant tau monomers into ThT-positive fibrils. Saijo et al. (2017) described the first tau RT-QuIC assay, which provided ultrasensitive and specific detection of the 3R tau deposits in *post-mortem* brain (at ≤billion-fold dilutions) and CSF from PiD patients. Not surprisingly, this assay used a 3R tau fragment (K19CFh) as a substrate. The addition of a second tau substrate (τ306) encompassing the 4R portion of the AD fibril enabled a new assay to detect tau aggregates composed of both 3R/4R tau in brain (≤10 billion-fold dilutions) from AD and CTE patients with low femtogram analytical sensitivity (Kraus et al., 2019). Further improvements in assay reliability were obtained using a single recombinant 3R tau substrate (K12CFh) (Metrick et al., 2020). This tau K12 RT-QuIC assay also provided a single assay for ultrasensitive detection and discrimination of both the 3R tau seeds of PiD and the 3R/4R seeds of AD and CTE patients. The discrimination was based on relative ThT fluorescent maxima. Recently, the application of tau RT-QuIC to 4R tauopathies (4R RT-QuIC), allowed the discrimination of disease-specific conformers with high sensitivity (≥∼2 femtograms) in brain tissues and CSF from PSP and CBD patients. The study showed for the first time an ability to detect tau 4R aggregates in *antemortem* CSF samples (Saijo et al., 2020), suggesting that with further improvements in sensitivity such an assay might become useful clinically for diagnosing tau 4R tauopathies.

## Conclusion

Over the last decade, encouraging progress in the development of new diagnostic techniques for prion disorders have been made. Results from many different laboratories have shown that prion RT-QuIC can be robust and reliable for prion disease diagnosis, and as such, is being widely implemented in clinical practice and added to official diagnostic criteria. The diagnostic sensitivity of prion RT-QuIC is especially good for most subtypes of the predominant human prion disease, i.e., sporadic CJD; however some rarer human prion diseases have been more difficult to detect using CSF specimens. More recently, extensive applications of αSyn RT-QuIC and closely related (i.e., αSyn PMCA) SAAs to specimens from various synucleinopathy patients have demonstrated unprecedented early *antemortem* diagnostic accuracy for these otherwise difficult-to-diagnose diseases. Among the synucleinopathies, MSA seeds have been difficult to detect in patients’ CSF with the initial αSyn RT-QuIC assays, but αSyn PMCA conditions have been adapted to yield higher sensitivities for MSA (Shahnawaz et al., 2020). RT-QuIC applications to tauopathies have also shown promising initial results in detecting and discriminating tau seeds in biospecimens with ultra-sensitivity. However, further development of tau RT-QuIC assays is required to detect tau seeds in diagnostic biospecimens collected from live patients consistently enough for routine clinical utility. Although not mentioned above, another nascent and potentially important type of RT-QuIC assay detects seeds of TDP-43 (Scialo et al., 2020), a protein that is found to be aggregated in the pathology of several neurodegenerative diseases such as types of amyotrophic lateral sclerosis and frontotemporal dementia. Further development and validation of TDP-43 RT-QuIC will also be needed to fully realize its applicability in clinical practice. Nonetheless, multiple RT-QuIC assays are providing attractive and increasingly validated tools for accurately identifying proteopathies in living patients, which in turn, potentiates early diagnosis as well as the development of treatments for these disorders.

## Author Contributions

SV provided the initial draft. All authors edited the manuscript.

## Conflict of Interest

BC and CO have patents pending that relate to various RT-QuIC assays. The remaining author declares that the research was conducted in the absence of any commercial or financial relationships that could be construed as a potential conflict of interest. The handling editor declared a past co-authorship with one of the authors BC.

## Publisher’s Note

All claims expressed in this article are solely those of the authors and do not necessarily represent those of their affiliated organizations, or those of the publisher, the editors and the reviewers. Any product that may be evaluated in this article, or claim that may be made by its manufacturer, is not guaranteed or endorsed by the publisher.
